# Genomic characterization of the endangered medicinal polypore agarikon (*Laricifomes officinalis* syn. *Fomitopsis officinalis*)

**DOI:** 10.1093/g3journal/jkag237

**Published:** 2026-08-25

**Authors:** Patrick I Bennett, Zolton J Bair, Alexander J Bradshaw, Paul Stamets

**Affiliations:** Department of Research and Development, Fungi Perfecti, LLC, PO Box 7634, Olympia, WA 98507, United States; Department of Research and Development, Fungi Perfecti, LLC, PO Box 7634, Olympia, WA 98507, United States; Biology Department, Lasry Center for Bioscience, Clark University, 15 Maywood Street, Worcester, MA 01610, United States; Department of Research and Development, Fungi Perfecti, LLC, PO Box 7634, Olympia, WA 98507, United States

**Keywords:** Polyporales, reference genome, comparative genomics, biosynthetic gene clusters, secondary metabolites, eburiko

## Abstract

Agarikon (*Laricifomes officinalis* syn. *Fomitopsis officinalis*) is an endangered fungus belonging to a unique lineage in the Polyporales (Basidiomycota) with a growing body of evidence supporting its medicinal value. In this study, we report the hybrid *de novo* assembly and annotation of the nuclear and mitochondrial genomes of *L. officinalis*, with a nuclear genome size of 28.76 Mb assembled across 66 contigs (51.96% GC content; BUSCO completeness of 99.4%) and a complete core mitochondrial genome size of 197.67 kb. Structural and functional annotation of the nuclear genome yielded 8,717 predicted genes including 8,604 protein-coding genes, with 287 genes in 27 candidate biosynthetic gene clusters. We characterized the mating type loci *matA* and *matB,* consistent with a tetrapolar mating system, and identified genes encoding key enzymes involved in triterpenoid and polyketide biosynthetic pathways that lead to the production of a diverse array of secondary metabolites. Additionally, we conducted maximum likelihood phylogenomic analysis to confirm the taxonomic position of *L. officinalis* among other species in the Polyporales using protein sequences for 730 shared BUSCO genes. This high-quality annotated genome of *L. officinalis* will serve as a foundation for further investigations into its evolutionary history, provide a reference for future population genomic analyses, and elucidate mechanisms underlying the synthesis of the bioactive compounds responsible for agarikon's wide-ranging medicinal benefits.

## Introduction

The polypore known colloquially as agarikon, eburiko, and “quinine conk” (*Laricifomes officinalis* (Vill.) Kot. & Pouzar syn. *Fomitopsis officinalis* (Vill.) Bondartsev & Singer) is a flagship species of old-growth forest ecosystems and is classified as Endangered on the International Union for Conservation of Nature (IUCN) Red List due to ongoing population decline resulting primarily from overharvesting and habitat loss occurring since the early 20th century (Kałucka and Svetasheva 2019; Girometta et al. 2021; Gilhen-Baker et al. 2022). Agarikon has been recorded across the temperate and boreal forests of the Northern Hemisphere, including North America, Eurasia, and North Africa, with a geographic distribution generally coinciding with those of its coniferous tree hosts, including but not limited to larch (*Larix* spp.), Douglas-fir (*Pseudotsuga menziesii*), cedar (*Cedrus* spp.), fir (*Abies* spp.), pine (*Pinus* spp.), spruce (*Picea* spp.), and hemlock (*Tsuga* spp.) (Kałucka and Svetasheva 2019).

*Laricifomes* represents a distinct lineage within the Polyporales (Binder et al. 2013; Ortiz-Santana et al. 2013; Liu et al. 2023; Spirin et al. 2024). Despite its previous taxonomic classification, phylogenetic analyses have shown that it does not form a monophyletic group with members of the genus *Fomitopsis* and thus is now classified as the only known member of the genus *Laricifomes* in a family with the proposed name of Laricifomitaceae (Ortiz-Santana et al. 2013; Han, M-L et al. 2016; Liu et al. 2023; Spirin et al. 2024). Two other species, *Laricifomes maire* G. Cunn. and *Laricifomes concavus* (Cooke) G. Cunn., were classified in the genus previously, though these are now considered members of the genus *Fomitopsis* (Cunningham 1950; Buchanan and Ryvarden 1988, 2000; Spirin et al. 2024). However, there is a lack of genetic data available to support a definitive taxonomic classification of these species in relation to *L. officinalis*. The current taxonomic position of *L. officinalis* in its own genus may reflect its evolutionary divergence from other known polypores, suggesting that it could possess unique genomic and ecological adaptations as well as potentially novel physiological and biochemical properties.

Wood decay fungi serve important roles in forest ecosystem processes such as carbon cycling and tree regeneration (Fukasawa 2021; Niego et al. 2023). Brown-rot fungi such as *L. officinalis* produce lignin-rich residues that contribute to the accumulation of organic matter in soils, increasing water-holding capacity and creating favorable microsites for seedling regeneration (Gilbertson 1980; Fukasawa 2021). As a heart rot fungus, agarikon also promotes the formation of cavities that serve as habitat for many wildlife species including small mammals and birds (Gilbertson 1980; Jackson and Jackson 2004; Fukasawa 2021). In addition to the many ecosystem services provided, *L. officinalis* also has a long history of use for medicinal and spiritual purposes (Gilbertson 1980; Blanchette et al. 1992).

The first written documentation of its use as medicine dates to the first century CE, when Dioscorides Pedanius wrote about the use of “agaricon” (presumed to be *L. officinalis* based on the accounts of later authors) to treat a variety of illnesses including indigestion, hernias, dysentery, jaundice, asthma, and tuberculosis (Buller 1914; Gilbertson 1980; Dioscorides 2000). *Laricifomes officinalis* has been so highly regarded from a pharmacological perspective that it historically was recommended for treatment of “all internal disorders” (Buller 1914; Gilbertson 1980; Dioscorides 2000). Agarikon was also used as medicine for centuries in North America, Europe, North Africa, and Asia to treat a wide range of ailments (Gilbertson 1980; Stamets 2005a; Grienke et al. 2014; Muszyńska et al. 2020; Gafforov et al. 2025).

Recently, a placebo-controlled clinical trial demonstrated that a combination of agarikon and turkey tail (*Trametes versicolor*) mycelia cultivated on brown rice (FoTv) was an effective adjunct to vaccination, both reducing side effects and potentiating more durable antibody generation (Saxe et al. 2026a), while a related clinical trial demonstrated FoTv's value as an antiviral treatment, significantly reducing the number and severity of COVID-19 symptoms (Saxe et al. 2026b). Additionally, a preclinical study has shown that agarikon mycelium alone has immunomodulatory potential, as evidenced by its significant effects on the expression of genes involved in innate immunity (Doar et al. 2026). Although numerous studies have demonstrated the medicinal value of agarikon, the precise mechanisms of action are understudied, and many of the observed benefits lack a connection to causative bioactive compounds or biosynthetic pathways.

Basidiomycetes, and polypores in particular, are known to produce a wide array of secondary metabolites with demonstrated bioactivities (Lin et al. 2017; Hassan et al. 2022; Du et al. 2024; Karunarathna et al. 2025; Liu et al. 2025). Biosynthetic genes underlying some of these secondary metabolites have been characterized, including the sesquiterpene synthase (STS) genes encoding enzymes involved in the production of structurally diverse bioactive terpenes (Chen et al. 2022; Dong et al. 2022; Hassan et al. 2022; Wang et al. 2023), as well as polyketide synthase (PKS) gene clusters encoding enzymes involved in the production of bioactive molecules such as laetiporic acid, orsellinic acid, and their derivatives (Yu et al. 2016; Gressler et al. 2021; Zhang et al. 2022, 2024).

*Laricifomes officinalis* is now being viewed in a modern pharmacological context, with burgeoning drug discovery research being undertaken to investigate agarikon's diverse array of bioactive molecules for potential applications in the treatment of cancer, viral and bacterial infections, respiratory ailments, and inflammation (Stamets 2005b; Hwang et al. 2013; Girometta 2019; Fijałkowska et al. 2020; Han et al. 2020; Muszyńska et al. 2020; Gafforov et al. 2025). Secondary metabolites produced by *L. officinalis* include lanostane triterpenoids with demonstrated antimicrobial and anti-inflammatory activities (Wu et al. 2004, 2009; Han et al. 2016, 2020), chlorinated coumarins with anti-tuberculosis activity (Hwang et al. 2013), as well as various sesquiterpenoids, sterols, indoles, polysaccharides, and phenolic compounds with antibacterial, anti-cancer, anti-inflammatory, and antifungal potential (Fijałkowska et al. 2020; Gafforov et al. 2025). However, the genes encoding the biosynthetic machinery involved in the production of these compounds have not been previously characterized.

Here we present the nuclear and mitochondrial genomes of *L. officinalis,* evaluating genome structure and content with an emphasis on secondary metabolite biosynthesis and synteny with closely related polypore fungi. These reference genomes will serve as a valuable foundational resource for further research into the ecology, evolutionary history, and pharmaceutical potential of this endangered fungus and will facilitate efforts to study and conserve the genetic diversity present across its populations globally.

## Materials and methods

### Sample collection, isolation, and DNA extraction

The *L. officinalis* isolate sequenced for this study (“F01”) was originally collected in 2001 from a large conk growing on Douglas-fir near Morton, Washington, United States. Hymenial tissue was sampled using a sterile cork borer and used to isolate a pure dikaryotic mycelium culture on malt extract agar (MEA) amended with 50 mg/L gentamycin sulfate. Live cultures were maintained on MEA in refrigerated slants and preserved for long-term viability in cryogenic storage (Zalesky et al. 2024). For this study, mycelial tissue for DNA extraction was grown in malt extract broth and harvested via filtration. Genomic DNA was extracted from mycelial tissue using the E.Z.N.A. Plant and Fungal DNA kit (Cat. No. D3485-01, Omega Bio-Tek, Norcross, Giorgia, United States) following manufacturer instructions.

### DNA sequencing

DNA extracts were sent to Novogene Corporation Inc. (Sacramento, California, United States) for library preparation and whole genome sequencing. To enable a hybrid *de novo* genome assembly, long reads were sequenced on the PromethION platform (Oxford Nanopore Technologies plc, Oxford, UK), while the NovaSeq 6000 platform (Illumina, Inc., San Diego, California, United States) was used to sequence 150-bp paired-end short reads.

Illumina sequencing libraries were constructed using the NEBNext Ultra II DNA Library Prep Kit (New England Biolabs Inc., Ipswich, Massachusetts, United States), including end repair, dA-tailing, and ligation of NEBNext adapters. Fragments were enriched using PCR with P5 and indexed P7 oligos. After libraries were purified, a Qubit fluorometer (Thermo Fisher Scientific, Waltham, Massachusetts, United States) was used to determine concentrations, and an Agilent 2100 bioanalyzer (Agilent Technologies Inc., Santa Clara, California, United States) was used to assess insert size. Quantitative real-time PCR was performed to determine effective concentration prior to pooling libraries.

For long-read sequencing, high molecular weight DNA was size-selected, end-polished, nick repaired, A-tailed, and adapters were ligated using the Oxford Nanopore Ligation Sequencing Kit 1D (Oxford Nanopore Technologies plc, Oxford, UK). After purification with AMPure XP beads (Beckman Coulter, Brea, California, United States), concentration was measured using a Qubit fluorometer and size distribution was assessed with a bioanalyzer before quantified libraries were pooled for sequencing.

Quality control performed by Novogene on sequence data yielded reads free of low-quality base calls and adapter sequences. Raw read quality was further evaluated with SeqKit v2.12.0 (Shen et al. 2024), Nanoplot v1.41 (De Coster and Rademakers 2023) for ONT reads, and fastqc v0.12.1 (Andrews 2010) for Illumina reads.

### Genome assembly

Hybrid *de novo* genome assembly was performed using MaSuRCA v4.1.4 (Zimin et al. 2013; Zimin et al. 2017) with both ONT long reads and Illumina short reads. Preliminary assembly quality assessments were performed using QUAST v5.3.0 (Mikheenko et al. 2018) and BUSCO v6.0.0 (Tegenfeldt et al. 2025) prior to segregating mitochondrial components from the nuclear genome.

Two of the MaSuRCA-assembled contigs were identified as being of mitochondrial origin based on size, read depth, and GC content (%), as well as homology with publicly available fungal mitochondrion genome sequences (assessed via BLAST + v2.17.0). Preliminary analysis of a putative core mitochondrial contig (221 kb) using minimap2 v2.30-r1287 revealed low ONT read coverage at each end, as well as two duplicated mitochondrial genes, suggesting an erroneous linear assembly. Both mitochondrial contigs were excised from the MaSuRCA assembly, and all subsequent analyses handled nuclear and mitochondrial genomes separately.

The core mitochondrial genome was independently reassembled from Illumina reads using GetOrganelle v1.7.7.1 (Camacho et al. 2009; Langmead and Salzberg 2012; Jin et al. 2020; Prjibelski et al. 2020), with the two MaSuRCA-assembled mitochondrial contigs as a reference and the “fungus_mt’ preset as seeds. Annotation was performed using MFannot (Lang et al. 2023) with genetic code “4 Mold, Protozoan, and Coelenterate Mitochondrial; Mycoplasma/Spiroplasma” (https://megasun.bch.umontreal.ca/apps/mfannot/). The core mitochondrial genome was visualized using OGDRAW v1.3.1 (Greiner et al. 2019). The remaining 32-kb contig, putatively of mitochondrial origin, was evaluated independently and annotated with a combination of MFannot and HHpred (Zimmermann et al. 2018).

Illumina reads mapping to the nuclear contigs were isolated and subsequently utilized to polish the MaSuRCA nuclear assembly contigs with Pypolca v0.4.0 (Zimin and Salzberg 2020; Bouras et al. 2024). The polished nuclear assembly was cleaned and sorted using Funannotate v1.8.17 (Palmer and Stajich 2020). RepeatModeler v1.0.11 (Smit and Hubley 2008) was used for *de novo* detection and annotation of repeats and transposable elements by referencing the Dfam database (Storer et al. 2021). The resulting repeat library was used to soft-mask the genome with RepeatMasker v4.1.2-pl (Smit et al. 2013).

TelomereSearch (McGowan 2021) was used to scan the nuclear genome contigs for at least five copies of conserved fungal telomeric motifs within 1 kb of either end while allowing for flexibility in the nucleotide repeat length.

### Structural annotation

Structural annotation was performed on the soft-masked assembly using Funannotate, which included *ab initio* gene prediction using BUSCO to identify conserved gene models for training AUGUSTUS (Stanke et al. 2006; Hoff and Stanke 2019), glimmerHMM, and SNAP, in addition to self-training with GeneMark-ES (Lomsadze et al. 2005; Ter-Hovhannisyan et al. 2008). Reference proteomes from *Fomitopsis schrenkii* (UP000015241) (Floudas et al. 2012), *Fomitopsis quercina* (syn. *Daedalea quercina*) (UP000076727) (Nagy et al. 2016), and *Rhodonia placenta* (syn. *Postia placenta*) (UP000194127) (Gaskell et al. 2017) were downloaded from UniProt and provided as hints to assist gene prediction. A predicted protein data set from *Taiwanofungus camphoratus* (syn. *Antrodia cinnamomea*), downloaded from NCBI GenBank (GCA_022598655.1) (Chen et al. 2022), was also included as protein evidence. The quality of structural annotations was assessed using BUSCO in protein mode with both the basidiomycota_odb12 and polyporales_odb12 databases. A diagrammatic representation of the nuclear genome including contig structure, gene density in 10-kb sliding windows, GC content (%), and repeat density in 10-kb sliding windows, was produced using the circlize package (v0.4.18) (Gu et al. 2014) in RStudio (v2025.9.1.401; Posit Team 2025) with R v4.5.1 (R Core Team 2025).

### Functional annotation

Following structural annotation, functional annotation was performed using InterProScan5 v5.77-108.0 (Jones et al. 2014), antiSMASH v8.0.4 (Blin et al. 2025), and Funannotate, along with eggNOG-mapper (EggNog v2.1.13) (Huerta-Cepas et al. 2019; Cantalapiedra et al. 2021). Other databases referenced by Funannotate for functional annotations included dbCAN v12.0 (CAZymes), Gene2Product v1.97, GO v2026-01-23 (gene ontology), MEROPS v12.5 (protease identification), PFAM v38.1 (protein families), and UniProt DB v2026_01 (protein homology). Signal peptides were identified using DeepSig (Savojardo et al. 2018).

### Phylogenomics

MycoTools v1.0.0 (Konkel and Slot 2023) was used to retrieve reference genomes from NCBI GenBank for a diverse group of 20 fungal species representative of the Polyporales (Liu et al. 2023). The reference genome of *Heterobasidion irregulare* TC 32-1 (NCBI Accession No. GCF_000320585) was included as an outgroup. The predicted proteome for each species was subjected to BUSCO analysis in protein mode with the polyporales_odb12 lineage data set. BUSCO_phylogenomics v20260305 was used to identify BUSCOs shared among these taxa and *L. officinalis* F01 and to construct a concatenated supermatrix alignment of amino acid sequences of the shared genes, along with a partition file. IQ-TREE v3.1.2 (Chernomor et al. 2016; Hoang et al. 2018; Wong et al. 2026) was used to reconstruct a maximum-likelihood phylogeny using the supermatrix alignment and ModelFinder Plus (Kalyaanamoorthy et al. 2017) was used to determine the best-fit substitution model for each partition. Partitions sharing the same substitution model were merged. Branch support was calculated from 1,000 ultrafast bootstrap replicates. The resulting maximum-likelihood tree was rooted on *H. irregulare* (Olson et al. 2012) and visualized using ggtree v3.16.3 (Yu et al. 2017; Xu et al. 2022) in RStudio.

### Synteny analyses

A synteny analysis was performed using SYNY v1.3.2 (Li 2018; Buchfink et al. 2021; Kille et al. 2023; Julian and Pombert 2024) to compare genome content, structure, and organization between the *L. officinalis* genome and a publicly available annotated reference genome for *Laetiporus sulphureus* 93-53 (NCBI GenBank, GCF_001632365.1) (Nagy et al. 2016) using all contigs larger than 500 kb from both genomes. To identify mating type loci in the *L. officinalis* F01 genome, the annotation file was queried for product names corresponding to genes such as mitochondrial intermediate peptidase (*mip*) for *matA* or *ste3* for the *matB* locus (James et al. 2013; Dong et al. 2022). Synteny among homologous genes in the mating-type gene clusters was evaluated using clinker v0.0.32 (Gilchrist and Chooi 2021) via the online CompArative GEne Cluster Analysis Toolbox (CAGECAT, v1.0) (van den Belt et al. 2023). To facilitate synteny analysis of biosynthetic gene clusters (BGCs) among related polypores, genomes of *Lae. sulphureus* 93-53 (GCF_001632365.1) and *T. camphoratus* V5 (GCA_022598655.1) were also evaluated using antiSMASH v8.0.4 (Blin et al. 2025). The *T. camphoratus* V5 genome annotations (GFF) retrieved from NCBI GenBank included multiple coding sequences associated with the same locus, requiring AGAT v1.6.1 (Dainat et al. 2026) to identify overlapping genes and retain only the longest isoform prior to synteny analysis with clinker. Target BGC regions were isolated and similarly submitted via CAGECAT for clinker analysis to produce plots to evaluate synteny/collinearity.

### Analyses of genes encoding sesquiterpene synthase enzymes

Sesquiterpene synthase (STS) genes in the agarikon genome were classified based on functional annotations, including Pfam and Interpro domains, as well as the presence of conserved amino acid motifs (Wang et al. 2023). Amino acid sequences of the 11 STS genes identified in the *L. officinalis* F01 genome were aligned with those of selected basidiomycete STS genes characterized via heterologous expression and representative of the four STS clades (Ichinose and Kitaoka 2018; Wu et al. 2022; Wang et al. 2023). The alignment was constructed using MAFFT v7.525 (Katoh et al. 2002; Katoh and Standley 2013) with the L-INS-i (−localpair) method and a maximum of 1,000 iterations for refinement. A maximum-likelihood phylogenetic reconstruction was performed using IQ-TREE v3.1.2 (Wong et al. 2026) with ModelFinder Plus (Kalyaanamoorthy et al. 2017) and 1,000 ultrafast bootstrap replicates (Hoang et al. 2018). The tree was rooted at the midpoint and visualized with ggtree in RStudio.

## Results and discussion

### Sequence data quality

Illumina sequencing produced a total of 23,292,072 reads comprising approximately 3.5 Gb, resulting in an average coverage depth of 121.5 × with 91.3% of bases exceeding a Q30 Phred score. After filtering to a minimum average read quality threshold of Q7, Oxford Nanopore data contained 115,538 reads (76.4% of total reads generated) with an average length of 4.8 kb, N50 read length of 7,726 bp, and a total of approximately 555 Mb, resulting in an average depth of approximately 19.3 × (Supplementary Tables 1 and 2).

### Assembly of the nuclear and mitochondrial genomes

The initial MaSuRCA assembly yielded 68 contigs with a total length of approximately 29 Mb and an N50 of 997.5 kb with the largest contig spanning more than 2.8 Mb. Although the average GC content of the total assembly was 51.74%, an examination of per-contig GC and Illumina mapped depth revealed the presence of two contigs that did not conform to the expected characteristics of nuclear genome content. The remaining 66 contigs representing the nuclear genome had GC content of 51.96%, average Illumina mapped depth of 79x, and average ONT mapped depth of 14.9x, for a total genome sequencing coverage of approximately 94x.

The larger of the two putative mitochondrial contigs was reconstructed by GetOrganelle to produce a complete circular mitochondrial genome 197,672 bp in length, with 26.4% GC content. The smaller of these two contigs was presumed to be of mitochondrial origin due to its lower GC content (28%) and higher Illumina mapped depth (754x), which differed substantially from the contigs representing the nuclear genome. This 32-kb linear contig contained six tandem copies of nearly identical nucleotide sequence. Although reminiscent of a mitochondrial plasmid, fluctuations in ONT read depth along with multiple sequence alignment of the subunits suggest this contig may have contained assembly artifacts arising from complex nested repeats; therefore, the tandem copy number should be interpreted with caution. Annotation of this contig with MFannot and HHpred identified the presence of an organellar DNA polymerase type B (DNA_pol_B_2; PF03175.19), with detected sequence features including coiled coil and transmembrane segments. While this contig has some characteristics of a mitochondrial plasmid, its true structure could not be fully resolved and the possibility that it contained assembly artifacts could not be disproved. Given the ambiguous nature of this contig, it was excluded from further analyses.

### Assembly quality

Downstream analyses treated the nuclear and mitochondrial genome assemblies separately. Pypolca polishing corrected 71 substitution errors and two indel errors in the nuclear genome. All subsequent analyses were performed using the polished nuclear genome assembly file. The nuclear assembly contained 66 contigs and a total length of 28.76 Mb with the largest contig being 2.84 Mb. The N50 was 997,481 bp, and overall GC content was 51.96% (Table 1, Supplementary Table 3). BUSCO analysis of the nuclear assembly with the basidiomycota_odb12 lineage dataset (*N* = 2,409 total BUSCO groups searched) indicated 2,395 complete BUSCOs (C = 99.4%); 2,379 complete and single copy (S = 98.8%); 16 complete and duplicated (D = 0.7%); one fragmented (F < 0.1%); and 13 missing (M = 0.5%). Analysis with the polyporales_odb12 lineage dataset (*N* = 4,690) indicated 4,626 complete BUSCOs (C = 98.6%); 4,592 complete and single copy (S = 97.9%); 34 complete and duplicated (D = 0.7%); one fragmented (F < 0.1%); and 63 missing (M = 1.3%).

**Table 1.** Assembly metrics for the *Laricifomes officinalis* F01 nuclear genome, with BUSCO completeness assessed using the basidiomycota_odb12 lineage dataset (*N* = 2,409).

Metric	Value
Genome size (bp)	28,757,392
Contigs	66
N50 (bp)	997,481
Largest contig (bp)	2,838,896
GC content	51.96%
Complete BUSCO	99.4%
Single-copy	98.8%
Duplicated	0.7%
Fragmented BUSCO	<0.1%
Missing BUSCO	0.5%

Two contigs in the nuclear genome assembly contained telomeric repeats within 1 kb of both ends, while 23 contigs contained telomeric repeats near one end. Although most assembled contigs do not represent full chromosomes, many contain at least one telomere.

### Structural annotation of the nuclear genome

Following RepeatModeler and RepeatMasker, 10.32% of the genome was soft-masked, with 7.41% consisting of long terminal repeat (LTR) elements (Supplementary Table 4). Four small RNAs were identified, accounting for 0.24% of the elements masked by RepeatMasker. Nearly 2% of the elements masked were listed as unclassified, suggesting the presence of species-specific transposable elements that did not match any known repeat families in the Dfam database. In general, regions with the highest density of repeats corresponded with areas of lower GC content and gene density (Fig. 1).

**Fig. 1.**
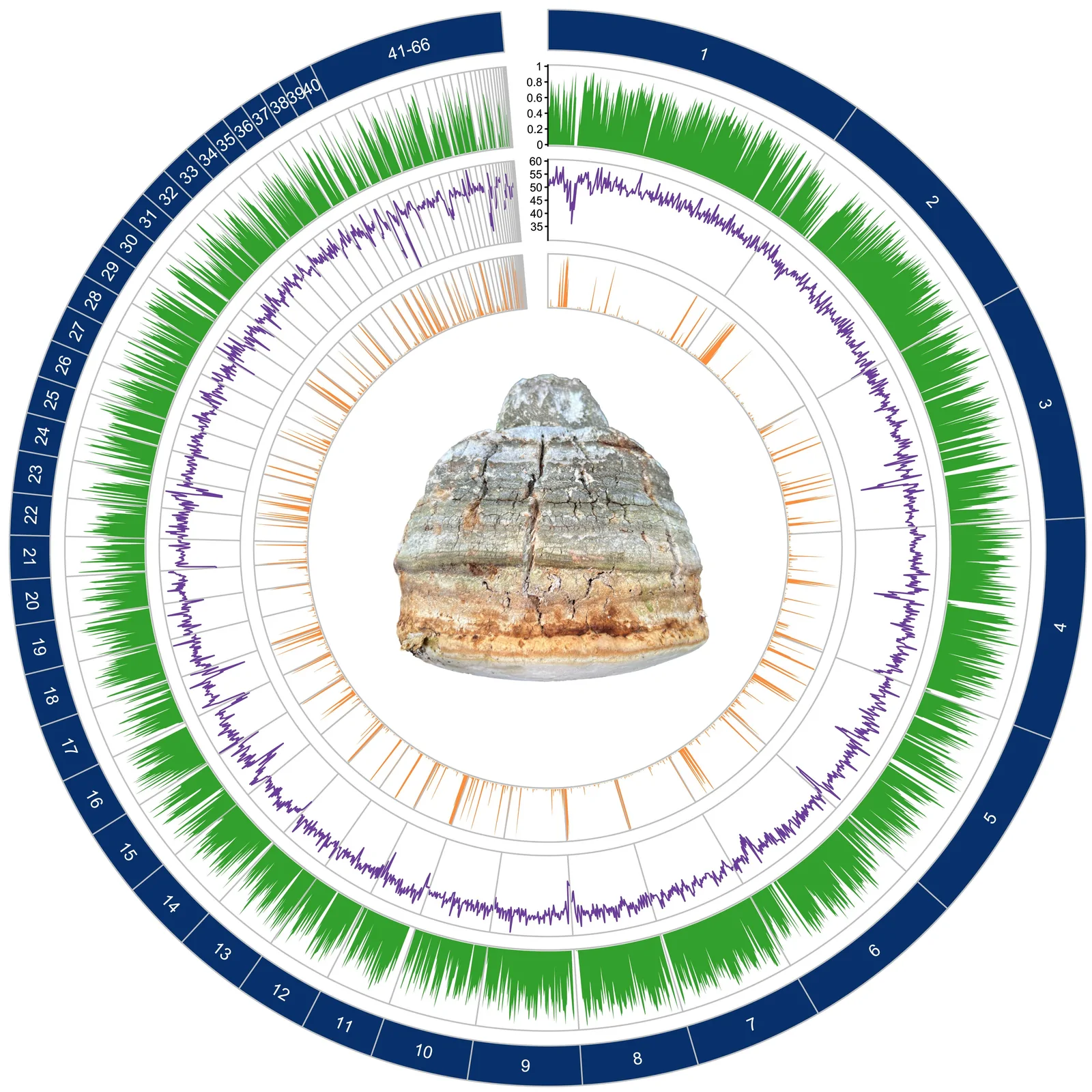
Diagrammatic representation of the nuclear genome of *Laricifomes officinalis* F01 with an image of an *L. officinalis* basidiocarp at the center. Tracks from outer to inner represent contigs (blue), gene density in 10-kb sliding windows (green), GC percentage (purple), and repeat density in 10-kb sliding windows (orange).

Structural annotation revealed that two nuclear contigs did not contain any gene content. These contigs consisted primarily of repeat content that was masked by RepeatMasker— one was approximately 33 kb with 80.8% repeat content (contig 58), while the other was approximately 10 kb with 88.0% repeat content (contig 65).

Structural annotation yielded 8,717 total gene models with 8,604 protein-coding genes and 113 tRNAs (Table 2). The BUSCO results for the predicted proteins from the basidiomycota_odb12 database (*N* = 2,409) showed 2,172 complete (C = 90.2%); 2,157 complete and single copy (S = 89.5%); 15 complete and duplicated (D = 0.6%); 121 fragmented (F = 5.0%); and 116 missing (M = 4.8%). For the polyporales_odb12 database (*N* = 4,690), the results showed 4,142 complete (C = 88.3%); 4,108 complete and single copy (S = 87.6%); 34 complete and duplicated (D = 0.7%); 231 fragmented (F = 4.9%); and 317 missing (M = 6.8%).

**Table 2.** Summary of nuclear genome annotations for *Laricifomes officinalis* F01.

Total genes	8,717
Number of predicted protein-coding genes	8,604
tRNA	113
Total carbohydrate active enzymes (CAZymes)	223
Auxiliary activities (AA)	39
Carbohydrate-binding modules (CBM)	2
Carbohydrate esterases (CE)	12
Glycoside hydrolases (GH)	115
Glycosyl transferases (GT)	52
Polysaccharide lyases (PL)	3
Secondary metabolites	
AntiSMASH regions	23
Candidate biosynthetic gene clusters	27
Terpene	15
Terpene-precursor	3
NRPS-like**^a^**	7
Type 1 PKS**^b^**	2
Total genes in biosynthetic gene clusters	287

^a^NRPS, nonribosomal peptide synthase.

^b^PKS, polyketide synthase.

### Annotation of the core mitogenome

Examination of the 197,672 bp circular assembly by GetOrganelle provided strong evidence that this represents the fully resolved core mitochondrial genome. Mitochondrion-specific annotation revealed the core protein-coding genes expected for a fungal mitogenome (Fonseca et al. 2021) interspersed with a total of 54 introns, including 2 copies of *atp6*, as well as *atp8*, *atp9*, *cob* (11 introns), *cox1* (23 introns), *cox2* (6 introns), *cox3* (2 introns), *nad1* (4 introns), *nad2* (1 intron), *nad3*, *nad4*, *nad4L*, *nad5* (7 introns), *nad6*, and *rps3*. While mitochondrial rRNA genes (*rns* and *rnl*) were detected, they were not fully annotated by MFAnnot due to ambiguous start and stop boundaries. Partial DNA polymerase genes (*dpo*) were also identified by MFAnnot but were annotated as likely pseudogenes introduced by plasmid integration. Furthermore, there were 24 tRNA genes and 91 unique open reading frames (ORFs) identified, with many representing homing endonuclease genes (HEGs) of the LAGLIDADG and GIY-YIG families (Supplementary Table 5). Numerous ORFs were associated with introns in coding genes, resulting in fragmentation of the gene sequences and inflation of the mitochondrial genome size (Fig. 2) (Fonseca et al. 2021; Feng et al. 2024).

**Fig. 2.**
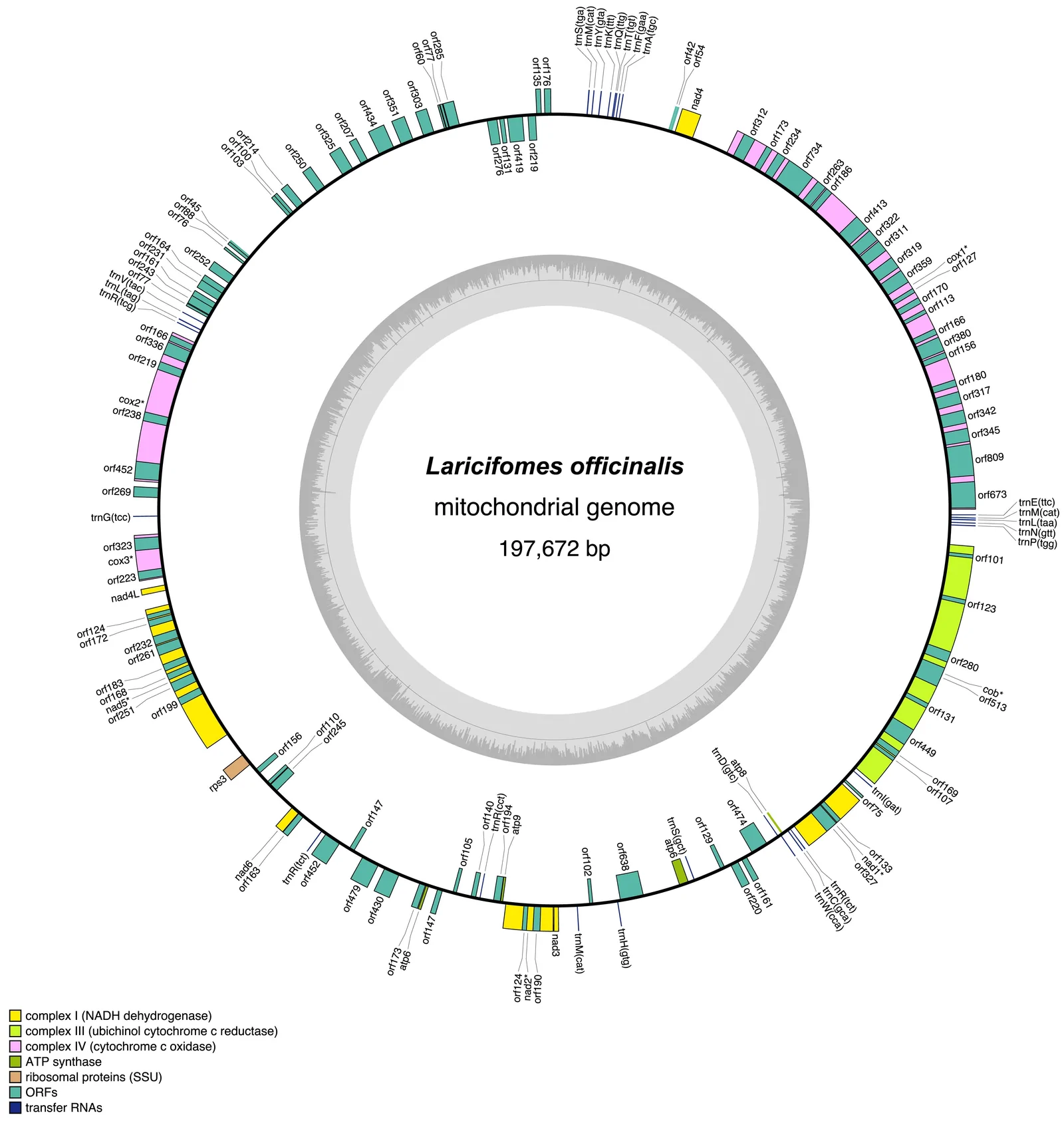
Diagrammatic representation of the complete circular mitochondrial genome of *Laricifomes officinalis* F01 showing protein-coding genes, ORFs, and tRNAs. Asterisks indicate genes that contain introns. The graph on the inner circle represents the GC content (%) with a line indicating the 50% level.

It appears that the proliferation of introns, in some cases mediated by the actions of HEGs, has contributed to significant expansion of the *L. officinalis* mitochondrial genome. Considerable intron loss and gain events have occurred in the evolutionary history of Polyporales, resulting in highly variable mitogenome sizes (Feng et al. 2024), with an examination of 12 species revealing a maximum of 36 introns in the 156 kb mitogenome of *Phlebia radiata* (Salavirta et al. 2014; Wang et al. 2020). At 197.7 kb, the *L. officinalis* mitogenome far exceeds the median for fungi (58 kb) and is currently among the largest mitochondrial genomes reported for basidiomycetes (Ahrendt et al. 2026).

### Functional annotation of the nuclear genome

Functional annotation of the nuclear genome resulted in the identification of 7,958 genes with eggNOG IDs, 5,500 genes with GO terms, 6,736 genes with InterPro domains, 2,501 specific gene products, 440 genes with signal peptides, and 223 genes encoding carbohydrate-active enzymes (CAZymes). Protein-coding genes were functionally annotated with terms corresponding to the Clusters of Orthologous Groups (COG) database, with 6,496 being assigned at least one COG term, including 1,821 with unknown functions (Supplementary Fig. 1). A total of 5,667 genes received at least one Pfam annotation, with a distribution of Pfam domains suggesting investments in signal transduction and stress response via protein kinases (PF00069, PF07714) and fungal-specific transcription factors (PF04082), as well as numerous MFS transporters (PF07690), including some embedded in BGCs which may be involved in the export of bioactive secondary metabolites (Supplementary Fig. 2). Additionally, 3,640 unique GO terms were assigned across 5,500 genes (Supplementary Fig. 3). A total of 287 genes occurred in 27 biosynthetic gene clusters (BGCs) across 23 regions identified by antiSMASH (Table 2). However, the Funannotate output lists 310 genes with antiSMASH annotations including 29 biosynthetic enzymes and 79 smCOGs. This discrepancy in the number of genes reported in BGCs is due to the fact that the Funannotate output often includes one or more flanking genes that are only partially within the regions defined by antiSMASH.

### Phylogenomics and comparative genomics

A total of 730 BUSCO genes (polyporales_odb12) were shared among all 22 taxa included in the phylogenomic analysis (Supplementary Table 6) and were subsequently incorporated into a concatenated supermatrix alignment of amino acid sequences, containing 412,969 sites, 178,895 of which were parsimony informative. Following ModelFinder, the partitions corresponding to these 730 genes were merged into 38 partitions for further analysis. The maximum-likelihood phylogenetic tree depicted a clade containing *Fomitopsis* species that were formerly included in genera such as *Daedalea*, *Antrodia*, *Piptoporus*, and *Rhodofomes* (Ortiz-Santana et al. 2013; Liu et al. 2023; Spirin et al. 2024), along with a subtending clade containing *Lae. sulphureus* and *L. officinalis* featuring strong statistical support (Fig. 3). Although these species may appear as sister taxa in our phylogenomic analysis, this taxonomic relationship should be interpreted cautiously due to the absence of intermediate taxa more closely related to *L. officinalis* (Liu et al. 2023).

**Fig. 3.**
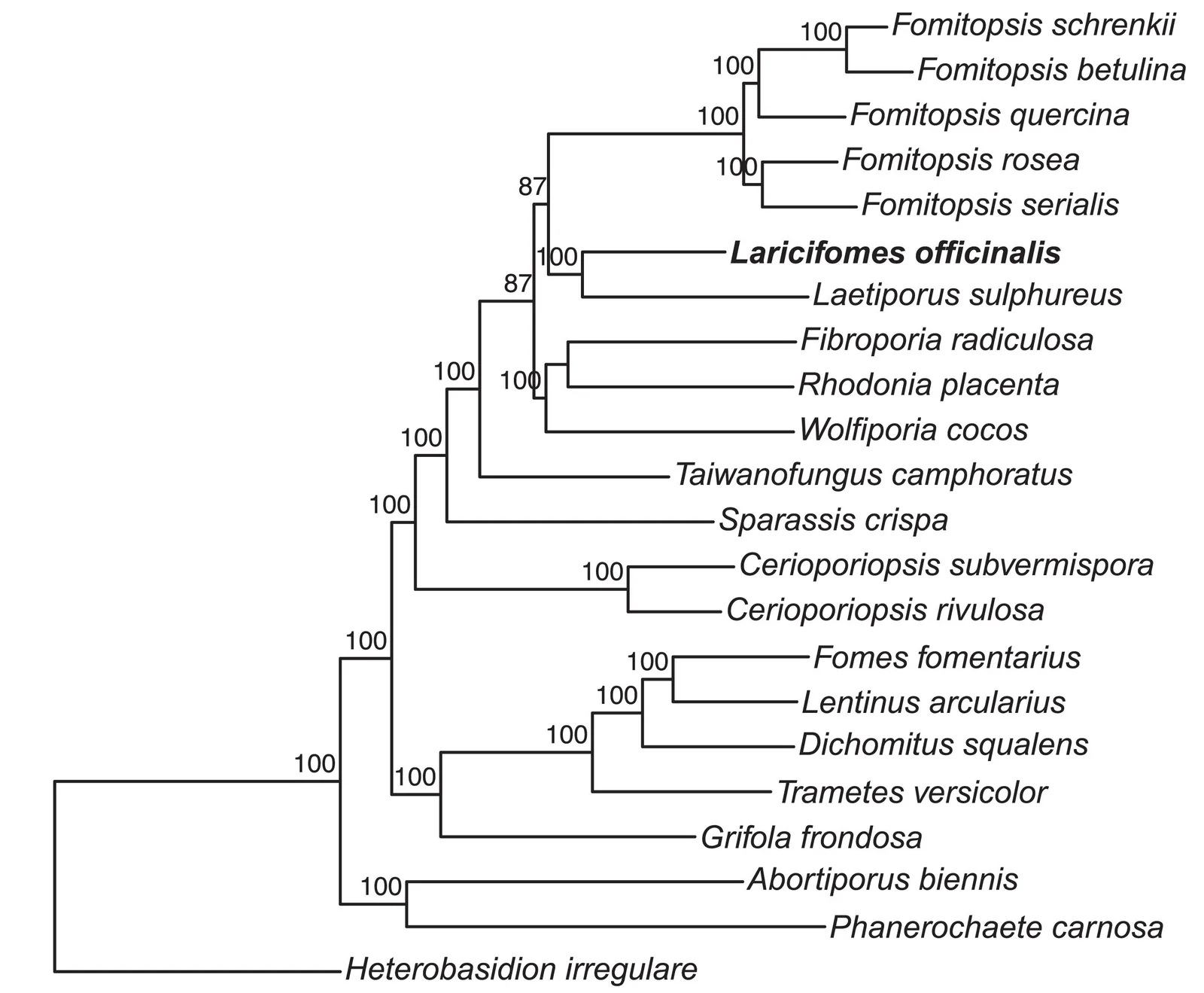
Maximum-likelihood phylogenetic tree constructed from a concatenated supermatrix alignment of amino acid sequences for 730 BUSCO genes shared among 22 species including *Laricifomes officinalis*, along with representative members of the Polyporales and an outgroup taxon in the Russulales. Branch support labels represent bootstrap values (%) from 1,000 ultra-fast bootstrap replicates.

Following the phylogenomic analysis, which revealed a close taxonomic relationship between *L. officinalis* and *Lae. sulphureus*, collinearity among the largest contigs of each of their genomes was assessed using a synteny plot (Fig. 4).

**Fig. 4.**
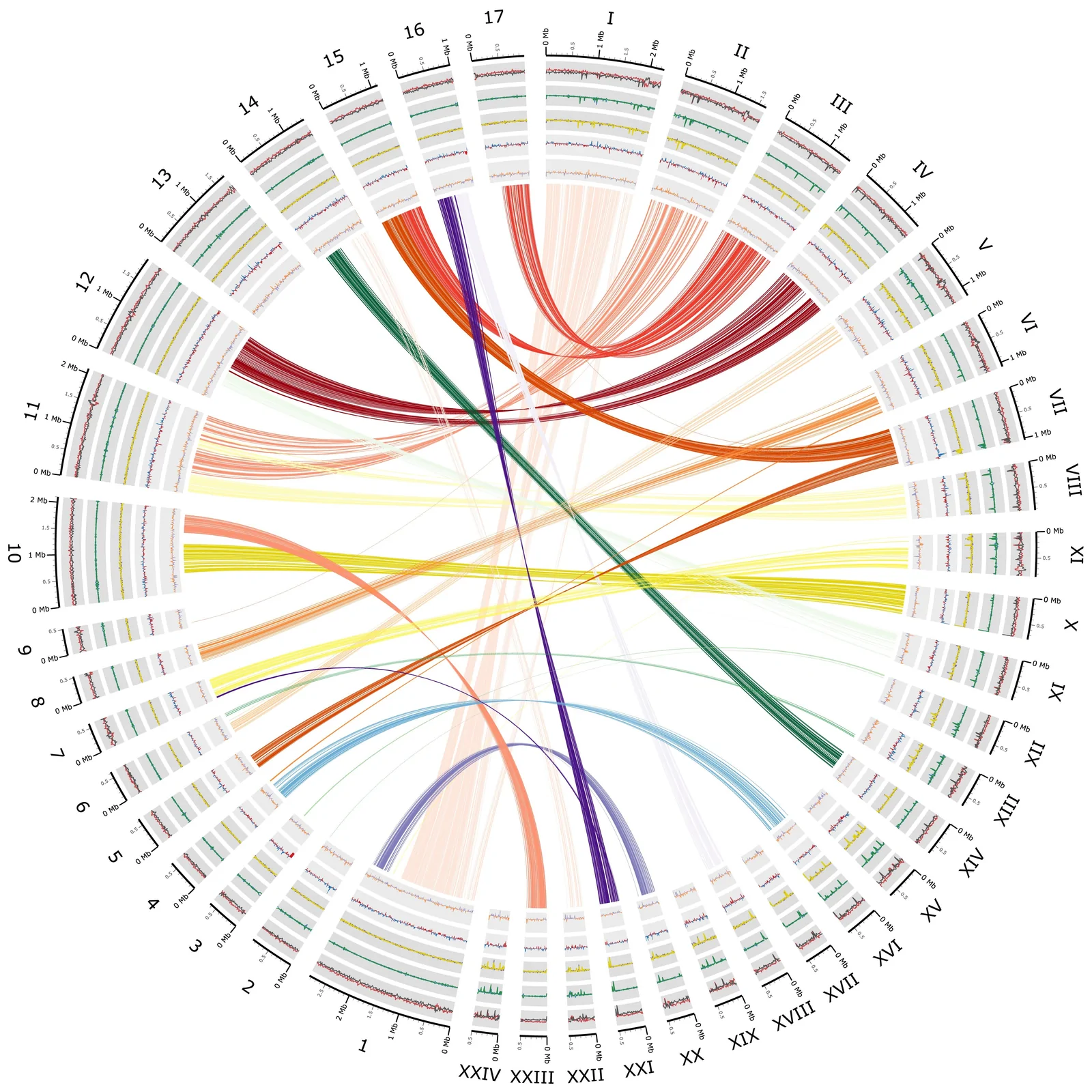
Synteny plot comparing the largest assembled genome contigs (>500 kb) from *Laricifomes officinalis* F01 (Arabic numerals) with those of *Laetiporus sulphureus* 93-53 (Roman numerals). Concentric subplots (from outer to inner) represent AT and GC nucleotide biases, GT and AC nucleotide biases, GA and CT nucleotide biases, GC skews, and AT skews. Colored ribbons indicate syntenic blocks shared among the contigs of each genome.

### Genome size

The agarikon genome is among the smallest reported for polypores in both assembly size and gene number. Nonetheless, the assembly showed high BUSCO completeness (99.4 and 90.2% for conserved basidiomycete genes and proteins, respectively), indicating a high-quality genome for a fungal lineage with limited characterization. Of the 93 genome assemblies representing 81 species available in the Joint Genome Institute Polyporales dataset (MycoCosm) (Grigoriev et al. 2014), only *Fibroporia radiculosa* has a smaller assembly (28.4 Mb; 9,262 genes) (Tang et al. 2012). In comparison, *L. officinalis* encodes just 8,717 genes in 28.8 Mb, exhibiting a gene density that is generally consistent with other polypore fungi. While the evolutionary and ecological drivers and consequences of this reduced genome size remain unclear, possible explanations include lineage-specific loss of non-essential genes or streamlining associated with its specialization toward slow growth on long-lived old-growth conifer hosts.

### Mating type genes

Gene regions corresponding to the tetrapolar mating type loci, *matA* and *matB*, were identified on contigs 2 and 5 of the nuclear genome, respectively. The *matA* locus displayed homologs of many elements expected for this highly conserved region, including homeodomain-encoding (HD) mating type genes (AC3AMU_001439, AC3AMU_001440), a glycosyl transferase family 8 gene (*glg2*) (AC3AMU_001435), a putative beta-flanking gene (*β-fg*) (AC3AMU_001438), and a mitochondrial intermediate peptidase gene (*mip* = octapeptidyl aminopeptidase, *oct1*) (AC3AMU_001441) (James et al. 2004, 2011, 2013; Dong et al. 2022; Zhang et al. 2022; Song et al. 2025). The *matB* locus comprises several pheromone mating factor G-protein coupled receptor (GPCR) genes (*ste3*) (AC3AMU_003118, AC3AMU_003122, AC3AMU_003123, AC3AMU_003124, AC3AMU_003125) and a major facilitator superfamily sugar transporter (*mfs1*) (AC3AMU_003119). Both mating loci were compared to those of *Lae. sulphureus* and *T. camphoratus* to evaluate synteny and collinearity across related species (Fig. 5). While there was broad collinearity among the genes present in the *matA* locus in *L. officinalis*, *T. camphoratus*, and *Lae. sulphureus*, the HD genes did not meet the minimum alignment sequence identity (0.30), suggesting diversification among these genes, as would be expected due to their involvement in mating type specificity (James et al. 2011).

**Fig. 5.**
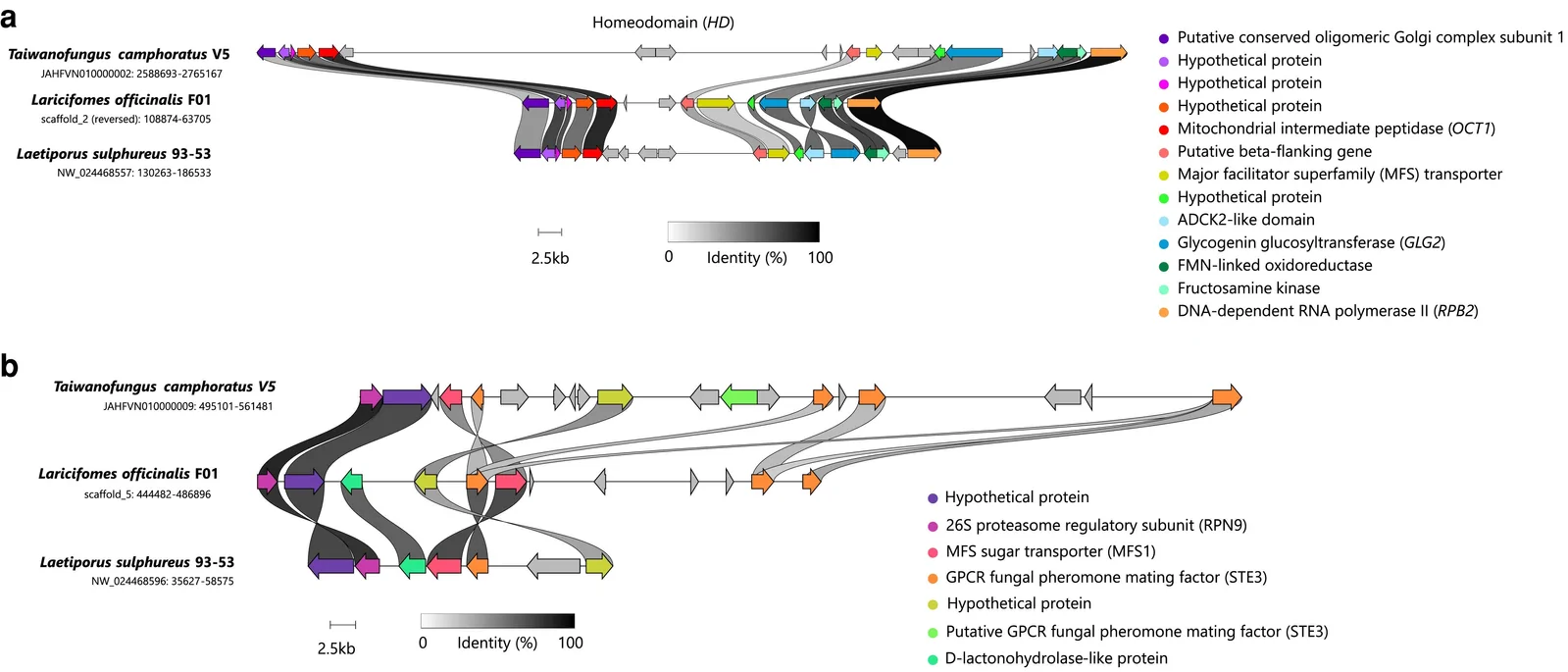
Synteny among gene clusters corresponding to the a) *matA* and b) *matB* loci from genome sequences of *Laricifomes officinalis* F01, *Laetiporus sulphureus* 93-53, and *Taiwanofungus camphoratus* V5.

### Carbohydrate-active enzymes

A total of 223 genes encoding a suite of carbohydrate active enzymes (CAZymes) were identified in the nuclear genome of *L. officinalis*. This included 115 genes encoding glycoside hydrolases (GH), 52 encoding glycosyl transferases (GT), 39 associated with auxiliary activities (AA), 12 encoding carbohydrate esterases (CE), 3 encoding polysaccharide lyases (PL), and 2 encoding carbohydrate-binding modules (CBM) (Table 2). The numbers of CAZymes observed in each of these classes are generally consistent with other brown rot fungi such as *T. camphoratus*, which exhibits a similar ecological life history (Chen et al. 2022).

### Terpenoid biosynthesis

The agarikon genome encodes numerous enzymes related to the biosynthesis of various terpenoids (Supplementary Table 7), to which the bioactivities and medicinal benefits of polypore fungi are often attributed (Lin et al. 2014, 2017; Saba et al. 2015; Kuo et al. 2016; Chen et al. 2022; Dong et al. 2022; Khalilov et al. 2022; Wang et al. 2023; Karunarathna et al. 2025). In particular, lanostane triterpenoids such as fomitopsins, officimalonic acids, sulphurenic acid, and eburicoic acid have been associated with medicinal effects including anti-tumor activity (Wu et al. 2004, 2009; Zhang et al. 2014, 2024, 2025; Han et al. 2016). Our analyses revealed several biosynthetic gene clusters (BGCs) involved in the mevalonate pathway and downstream triterpenoid synthesis, crucial to the production of ergosterol as well as secondary metabolites. While none of the BGCs in *L. officinalis* were functionally characterized as a part of this study, their putative functions have been inferred by comparison to those with known functions.

Specifically, we identified a BGC encoding a farnesyl pyrophosphate (FPP) synthase (*ERG20*, AC3AMU_002446) and two BGCs encoding enzymes responsible for the dimerization of FPP into squalene, a bifunctional FPP farnesyltransferase/squalene synthase (*ERG9*, AC3AMU_005648) and a putative squalene synthase gene (AC3AMU_002051). Although the epoxidase (*ERG1*, AC3AMU_003390) required to convert squalene into oxidosqualene was found outside of a BGC, we also characterized a distinct BGC anchored on the lanosterol synthase gene (*ERG7*, AC3AMU_007627) (Fig. 6a), which encodes an oxidosqualene cyclase responsible for converting oxidosqualene into lanosterol, an essential precursor for the production of diverse lanostane triterpenoids (Lin et al. 2015; Gressler et al. 2021; Zhang et al. 2025).

**Fig. 6.**
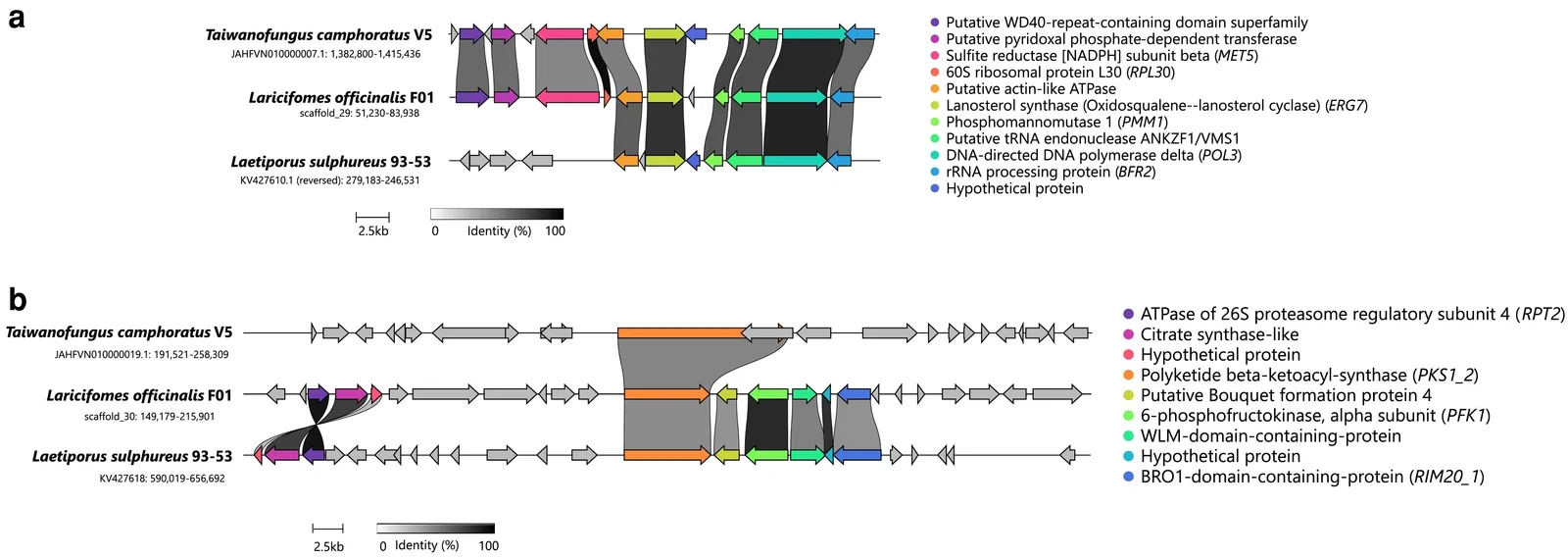
Synteny among secondary metabolite genes in biosynthetic gene clusters (BGCs) from the genomes of *Laricifomes officinalis* F01, *Laetiporus sulphureus* 93-53, and *Taiwanofungus camphoratus* V5. a) Genes encoding enzymes involved in the lanostane triterpenoid pathways, anchored on lanosterol synthase (ERG7). b) Genes in the BGC encoding a non-reducing PKS (NR-PKS) representing a putative homolog of an orsellinic acid biosynthesis cluster, anchored on the PKS gene.

We also identified a gene encoding delta(24)-sterol C-methyltransferase (*ERG6,* AC3AMU_008380), a key enzyme involved in the production of ergosterol and certain lanostane triterpenoids (Dong et al. 2022; He et al. 2025; Zhang et al. 2025). The predicted amino acid sequence shows significant homology to a steroid methyltransferase (SMT) enzyme identified from metabolomic and transcriptomic analyses of *T. camphoratus* and may be functionally equivalent, with one proposed function as a catalyst of dehydrosulphurenic acid biosynthesis (Zhang et al. 2025). Others suggest this enzyme may catalyze the production of a key intermediary in eburicoic acid biosynthesis by *Lae. sulphureus* (Dong et al. 2022).

### Sesquiterpenes

Numerous additional terpene-related BGCs were identified (Supplementary Table 8), suggesting a rich genomic repertoire for the biosynthesis of complex and diverse terpenoids. Among these, three BGCs contained genes encoding sesquiterpene synthases in basidiomycete STS Clade I (Fig. 7), which catalyze the production of compounds such as α-muurolene and cadinols via C1,10 cyclization of FPP (Ichinose and Kitaoka 2018; Nagamine et al. 2019; Gressler et al. 2021; Wu et al. 2022; Wang et al. 2023). The core biosynthetic gene in antiSMASH region 6.2 (AC3AMU_003641) was annotated as sesquiterpene synthase 3 (STS03) and is homologous to PpSTS03, an enzyme identified from *R. placenta* that catalyzes the production of β-elemene, γ-cadinene, δ-cadinene, and α-cadinene (Ichinose and Kitaoka 2018). The amino acid sequences corresponding to these enzymes share conserved binding site motifs including DDWSD and NDILSYNRE (Wang et al. 2023) and grouped together with strong support (100%) on the phylogenetic tree constructed with amino acid sequences from diverse basidiomycete STS genes. Similarly, antiSMASH region 13.1 contained an STS gene (AC3AMU_005810), which was annotated as *Agr3*, a homolog of the gene from *Cyclocybe* (= *Agrocybe*) *aegerita* encoding an enzyme that catalyzes the production of α-muurolene, δ-cadinene, and γ-muurolene (Zhang et al. 2020; Orban et al. 2021; Wang et al. 2023). The amino acid sequences of these genes also share conserved binding site motifs including DNISD and NDIFSYNVE and grouped together on the STS phylogeny, along with functionally similar enzymes from other basidiomycetes including Cop3, Omp3, and PoSTS03 (Wang et al. 2023) (Fig. 7). A third STS gene encoding an enzyme in Clade I, AC3AMU_007975, was identified in antiSMASH region 33.2. This gene was annotated as STS02 and is a homolog of PpSTS02 (= PpSTS01), which catalyzes the production of α-copaene, β-elemene, β-copaene, γ-muurolene, α-muurolene, and δ-cadinene (Ichinose and Kitaoka 2018). The amino acid sequences corresponding to these genes also shared binding site motifs DNISD and NDIFSYNVE (Wang et al. 2023) and grouped together with strong statistical support in the STS phylogeny.

**Fig. 7.**
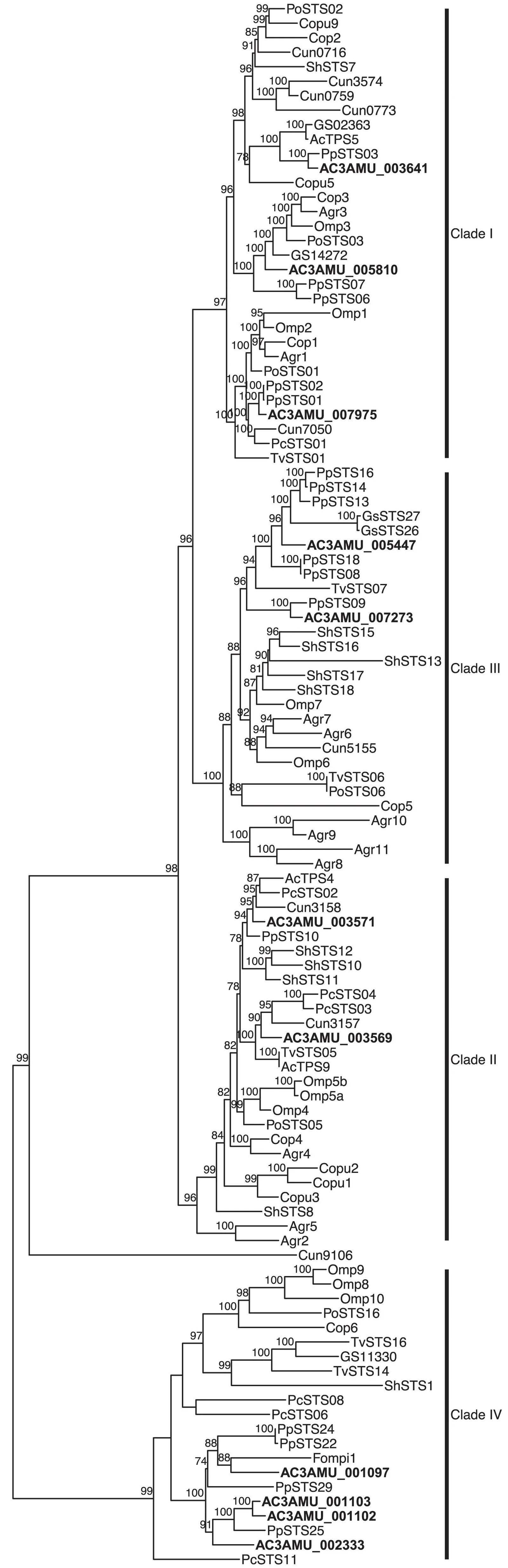
Maximum-likelihood phylogenetic tree constructed using amino acid sequences of previously characterized basidiomycete sesquiterpene synthase (STS) genes, along with those of the 11 STS genes identified from the genome of *Laricifomes officinalis* F01 (shown in bold), rooted at the midpoint. Node labels represent support values (> 70%) from 1,000 ultrafast bootstrap replicates. Clade labels correspond to the functional groupings of enzymes described in Wang et al. (2023), Wu et al. (2022), and Gressler et al. (2021).

AntiSMASH region 6.1 was identified as a terpene biosynthesis cluster containing two distinct STS genes annotated as STS10 (AC3AMU_003569 and AC3AMU_003571), which exhibited conserved amino acid motifs DEXSD and NDVYSYNME (Wang et al. 2023) and grouped with Clade II STS enzymes in the STS phylogeny (Fig. 7). These enzymes, such as PpSTS10 from *R. placenta* and AcTPS4 from *T. camphoratus*, generally catalyze the production of cadinenes via C1,10 cyclization of nerolidyl diphosphate (NPP) (Ichinose and Kitaoka 2018; Nagamine et al. 2019; Gressler et al. 2021; Wu et al. 2022; Wang et al. 2023). While AC3AMU_003571 clustered in a clade containing PpSTS10, AcTPS4, PcSTS02, and Cun3158 in the STS phylogeny, AC3AMU_003569 grouped more closely with AcTPS9, TvSTS05, Cun3157, PcSTS03, and PcSTS04. These enzymes catalyze the production of a diverse group of compounds including cadinenes, cubebenes, cubebols, copaenes, farnesols, farnesenes, germacrene D, muurolenes, and sibirene, among others (Ichinose and Kitaoka 2018; Nagamine et al. 2019; Gressler et al. 2021; Wu et al. 2022; Wang et al. 2023) (Supplementary Table 8).

Two BGCs—antiSMASH regions 11.1 and 25.1—contained genes encoding Clade III STS enzymes including AC3AMU_005447, encoding a putative homolog of PpSTS14 with shared motifs DEYTD and NDIASYNKE, and AC3AMU_007273, encoding an enzyme homologous to PpSTS09 with shared motifs DEYSD and NDMLSXNVE (Ichinose and Kitaoka 2018; Wang et al. 2023). In the STS phylogeny, the amino acid sequence of AC3AMU_007273 grouped with PpSTS09 with 100% bootstrap support, while AC3AMU_005447 grouped with a more diverse clade that included PpSTS14, along with other enzymes (Fig. 7). Clade III STS enzymes catalyze a C1,11 cyclization of FPP to produce complex tricyclic backbones such as pentalenene and Δ^6^-protoilludene (Ichinose and Kitaoka 2018; Gressler et al. 2021; Wu et al. 2022; Wang et al. 2023). Heterologous expression of PpSTS14 yielded pentalenene and caryophyllene as products, while PpSTS09 yielded an unidentified sesquiterpene (Ichinose and Kitaoka 2018). Collectively, STS enzymes in this clade are among the most important from a pharmaceutical perspective, as they can catalyze the production of a wide array of secondary metabolites with potential antitumor, antibiotic, and anti-inflammatory activities (Gressler et al. 2021).

### Trichodiene synthase homologs

AntiSMASH region 2.1 contained three STS genes including AC3AMU_001097 along with two distinct copies of the monoterpene synthase gene STS25 (AC3AMU_001102 and AC3AMU_001003). AntiSMASH region 3.2 contained a homologous gene, AC3AMU_002333. Despite minor variations in the amino acid motifs among these four genes present in the *L. officinalis* F01 genome, each featured trichodiene synthase (TRI5) domains based on Pfam (PF06330) and InterPro (IPR024652) annotations, as well as the presence of conserved amino acid motifs, including the Mg^2+^ binding site (DDXXX), a pyrophosphate and ion chelation region (NDXXSFYKEEL), and an FPP-binding site (XXRYRL) (Zhang et al. 2024). The amino acid sequences corresponding to AC3AMU_001102, AC3AMU_001003, and AC3AMU_002333 grouped with PpSTS25 in Clade IV in the STS phylogeny (Fig. 7) suggesting that they are homologs of PpSTS25 (Ichinose and Kitaoka 2018). The amino acid sequence of AC3AMU_001097 grouped with that of Fompi1, originally described from a North American isolate of *F. pinicola*, which was later determined to be *F. mounceae* (Wawrzyn et al. 2012; Haight et al. 2019; Spirin et al. 2024). These Clade IV STS enzymes catalyze a C1,6 cyclization of NPP to produce compounds such as α-cuprenene (Fompi1), as well as monoterpenes such as myrcene, and linalool (PpSTS25) (Ichinose and Kitaoka 2018; Gressler et al. 2021; Wu et al. 2022; Wang et al. 2023; Cong et al. 2024).

Trichodiene synthases catalyze the cyclization of FPP to the bicyclic sesquiterpene trichodiene (Wang et al. 2023), but divergent evolution has yielded TRI5-like enzymes with potential to biosynthesize other terpenes (Cong et al. 2024). Zhang et al. (2024) identified six of these *Tri5*-like genes in the genome of *Taiwanofungus gaoligongensis* and demonstrated their homology to proteins in *Sparassis crispa* and *Trametes versicolor*. Similarly, all eight predicted monoterpene synthases encoded in the *Lae. sulphureus* genome were homologous to PpSTS25 from *R. placenta* (Dong et al. 2022). These enzymes may represent trichodiene synthase-like sesquiterpene synthases (TDTSs), which can catalyze the production of diverse monoterpenes and sesquiterpenes (Cong et al. 2024).

Additional BGCs identified by antiSMASH included two associated with the production of terpene precursors, each encoding putative FPP synthase-like polyprenyl synthetases (AC3AMU_005238 and AC3AMU_006708). Another BGC involved in terpene biosynthesis included a haloacid-dehalogenase-like hydrolase gene clustered with two CYP450 monooxygenase genes and a short-chain dehydrogenase/reductase (SDR). Collectively, these results suggest that *L. officinalis* has the potential to produce a structurally diverse collection of terpenoid compounds.

### Polyketide biosynthesis

One large region near the edge of contig 28 was predicted by antiSMASH to contain a type 1 polyketide synthase (T1PKS) BGC amid three neighboring NRPS-like clusters (Supplementary Table 7), although it is unclear whether the apparent overlap between these reflects a functionally integrated biosynthetic region or low-confidence boundary prediction. This T1PKS cluster centered on a highly reducing beta-ketoacyl synthase (*pks1_1,* AC3AMU_007588) includes associated genes predicted to encode an AMP-dependent acyl ligase (AC3AMU_007594), a crotonyl-CoA reductase (AC3AMU_007593), a flavin-dependent halogenase (AC3AMU_007590), a Hotdog-fold thioesterase (AC3AMU_007585), multiple CYP450s (AC3AMU_007582, AC3AMU_007587, AC3AMU_007589), multiple MFS1 proton-linked monocarboxylate transporters (AC3AMU_007580, AC3AMU_007581, AC3AMU_007586, AC3AMU_007595), and an intermembrane transporter (AC3AMU_007591). Additional genes in the adjacent predicted NRPS-like clusters similarly encode AMP-dependent synthetase/ligases, additional CYP450s, and MFS1 transporters, as well as a D-lactate ferricytochrome c oxidoreductase (DLD1_3, AC3AMU_007579), an acyl-CoA dehydrogenase/oxidase (AC3AMU_007578), and a GMC oxidoreductase (AC3AMU_007572).

The presence of a putative flavin-dependent halogenase (AC3AMU_007590) in this T1PKS cluster raises the possibility that this region might contribute to the biosynthesis of chlorinated coumarins, which were previously discovered in the same *L. officinalis* isolate (F01) during an investigation of agarikon's antibiotic activity against *Mycobacterium tuberculosis* (Hwang et al. 2013). However, while the halogenase features a tryptophan fold commonly associated with aromatic substrate halogenation, the PKS (AC3AMU_007588) encodes a typical highly reducing PKS (HR-PKS) with a KS-AT-DH-KR-(NAD) domain architecture, which generally indicates aliphatic (rather than aromatic) polyketide synthesis. Consistent with most fungal HR-PKSs (Chooi and Tang 2012), this PKS also lacks a C-terminal reductase or thioesterase domain, indicating that polyketide chain release and downstream processing are likely mediated by trans-acting enzymes encoded in the region. In addition to the adjacent Hotdog-fold thioesterase (AC3AMU_007585) which could mediate the primary chain release, a pair of functionally equivalent adenylation enzymes with A-PP-TD domain architecture (AC3AMU_007594, AC3AMU_007605) could provide an alternative or subsequent route for additional post-PKS tailoring of metabolites produced by this BGC.

Together, these trans-acting enzymes provide a plausible mechanism for the decoupling of initial polyketide chain assembly and final backbone formation, potentially explaining how a single HR-PKS could support the production of multiple secondary metabolites with diverse structures, including aromatic compounds. Furthermore, oxidative transformations by the numerous CYP450s and redox enzymes in the region could also mediate aromatic cyclization of the following aliphatic polyketide synthesis, analogous to noncanonical fungal biosynthetic mechanisms in which reduced polyketides are converted to aromatic products through extensive post-PKS oxidative remodeling (Liu et al. 2019). Although the enzymes encoded within this T1PKS BGC are consistent with the formation, oxidative tailoring, potential halogenation, and export of polyketides, the specific enzymatic steps and metabolic product(s) remain speculative; furthermore, any potential functional involvement of the adjacent NRPS-like clusters remains to be determined.

### Non-reducing polyketide synthases

A second T1PKS BGC identified by antiSMASH contains a beta-ketoacyl synthase (*pks1_2*, AC3AMU_007745) (Supplementary Table 7), encoding a non-reducing PKS (NR-PKS) with the canonical domain structure SAT-KS-AT-PT-CP-CP-TE (Gressler et al. 2021). A homology search with BLASTp, along with synteny analysis via clinker (Fig. 6b), suggests this PKS is a putative homolog of PKS63787, an orsellinic acid synthase confirmed to be involved in the biosynthesis of antroquinonol meroterpenoids, benzenoids, and benzoquinones in *T. camphoratus* (Yu et al. 2016; Chou et al. 2017; Gressler et al. 2021; Kuang et al. 2021; Zhang et al. 2024). The *T. camphoratus* antroquinonols have been studied extensively, with substantial preclinical evidence demonstrating their anti-cancer and anti-inflammatory effects achieved through multiple pathways (Kuang et al. 2021), including by inhibiting nitric oxide, demonstrated following treatment of LPS-induced macrophages with multiple antroquinonols derived from the mycelium (Yang et al. 2009).

Homologs of this NR-PKS cluster were previously identified from the genomes of *T. gaoligongensis*, *T. camphoratus*, *Lae. sulphureus*, *Fomitopsis palustris*, and *F. quercina* (Zhang et al. 2024). The NR-PKS cluster identified in the genome of *L. officinalis* F01 is structurally similar to those of *Lae. sulphureus*, *F. palustris*, and *F. quercina*, which, unlike *Taiwanofungus* spp., all contain genes encoding modifying proteins including a 6-phosphofructokinase (AC3AMU_007747), a WLM-domain-containing-protein (AC3AMU_007748), and BRO1-domain-containing-proteins (RIM20_1, AC3AMU_007750 and RIM20_2, AC3AMU_007751) in addition to the core PKS gene (Zhang et al. 2024). However, in the *L. officinalis* genome, this cluster lacks the CYP450 genes that are present in the homologous clusters in *Lae. sulphureus* (Fig. 6b) and *F. palustris* (Zhang et al. 2024).

In addition to the terpene synthases, HR-PKS, and NR-PKS clusters described above, other terpene synthases and NRPS-like clusters were also identified by antiSMASH (Supplementary Table 7). These warrant further investigation as to their identity and homology with other fungal secondary metabolite clusters identified previously. Despite the fact that *L. officinalis* has a rather compact genome by comparison to other fungi in the Polyporales, an impressive diversity of secondary metabolite BGCs are encoded in its genome, though with fewer PKS and terpene clusters than are generally observed in the larger genomes of other polypores (Supplementary Table 9) (Chen et al. 2022; Dong et al. 2022; Zhang et al. 2022; Zhang et al. 2024; Song et al. 2025). However, additional PKS genes in the *L. officinalis* genome (AC3AMU_003055 and AC3AMU_003056) were not identified by antiSMASH as being part of a BGC, whereas a putative homolog was included in the antiSMASH output produced for this study using the *T. camphoratus* V5 genome. This suggests that the comparisons of the number and diversity of BGCs among *L. officinalis*, *Lae. sulphureus*, and *T. camphoratus* may not be reflective of the full suite of secondary metabolic enzymes encoded in the agarikon genome. While some of the identified BGCs have similarities to those described from other fungi, others appear to be potentially novel variants that may allow *L. officinalis* to produce a unique suite of secondary metabolites to which agarikon's numerous demonstrated medicinal benefits may be attributable.

## Conclusions

Here we present highly contiguous and extensively annotated nuclear and mitochondrial genomes of agarikon (*Laricifomes officinalis*), an endangered fungus representing a unique evolutionary lineage. In addition to resolving the core mitochondrion genome, potentially one of the largest among polypores, annotation of the relatively small 28.76 Mbp nuclear genome revealed 8,717 total genes, including 8,604 protein-coding genes. A genome-wide BUSCO phylogeny clarified agarikon's taxonomic position within the Polyporales, confirming that it does not form a monophyletic group with members of the genus *Fomitopsis*. While comparative genomic analyses revealed its affinity to *Laetiporus sulphureus*, it also showed similarities to *Taiwanofungus camphoratus* despite its more distant evolutionary relationship. Characteristics of both mating loci (*matA* and *matB*) were consistent with the tetrapolar mating system expected for a polypore fungus. Despite having one of the smallest nuclear genomes among fungi in the Polyporales, the *L. officinalis* genome encodes 287 genes in 27 candidate biosynthetic gene clusters with the potential to produce a diverse array of terpenoids, polyketides, and other bioactive compounds. These results serve as a valuable resource for targeted analytics and could lead to the discovery of potentially novel compounds that may have medicinal value. Future investigations will require functional validation via heterologous expression or targeted gene knockouts to confirm these findings and further elucidate the diversity of secondary metabolites produced by *L. officinalis.* Creating a robust reference genome for *L. officinalis* is a foundational step for understanding the biology of this unique fungus, which will enable future population genomics studies and facilitate functional characterization of secondary metabolites underlying the medicinal benefits of agarikon that have been reported for centuries.

## Supplementary Material

jkag237_Supplementary_Data

## Data Availability

All genomic data and associated metadata have been deposited in NCBI GenBank under BioProject PRJNA1460884 and BioSample SAMN57657796. Sequence data have been deposited in the Sequence Read Archive (SRA) with accession numbers SRR38352409 (ONT long reads) and SRR38352408 (Illumina short reads). For the nuclear genome, the Whole Genome Shotgun project has been deposited at DDBJ/ENA/GenBank under the accession JCANXR000000000. The version described in this paper is version JCANXR010000000. The associated nuclear genome assembly has been deposited in GenBank with accession number GCA_059492235.1 and the annotated mitochondrial genome assembly has been deposited in GenBank with the accession number PZ428821. All software code for reproducing the analyses and figures presented herein is publicly available in the following GitHub repository: https://github.com/Patrick-Bennett/Agarikon-Genomics. Supplemental material available at G3 online.
