# Supplementary material for: Genomic characterization of the endangered medicinal polypore agarikon (Laricifomes officinalis syn. Fomitopsis officinalis)
Source: G3 (Bethesda). 2026 Aug 25;16(11):jkag237. doi: 10.1093/g3journal/jkag237 (PMC13732445; doi:10.1093/g3journal/jkag237)
Supplement: Supplementary file 1 [file jkag237_supplementary_data.zip › Supplementary_Figure_3_G3-2026-407116.pdf]

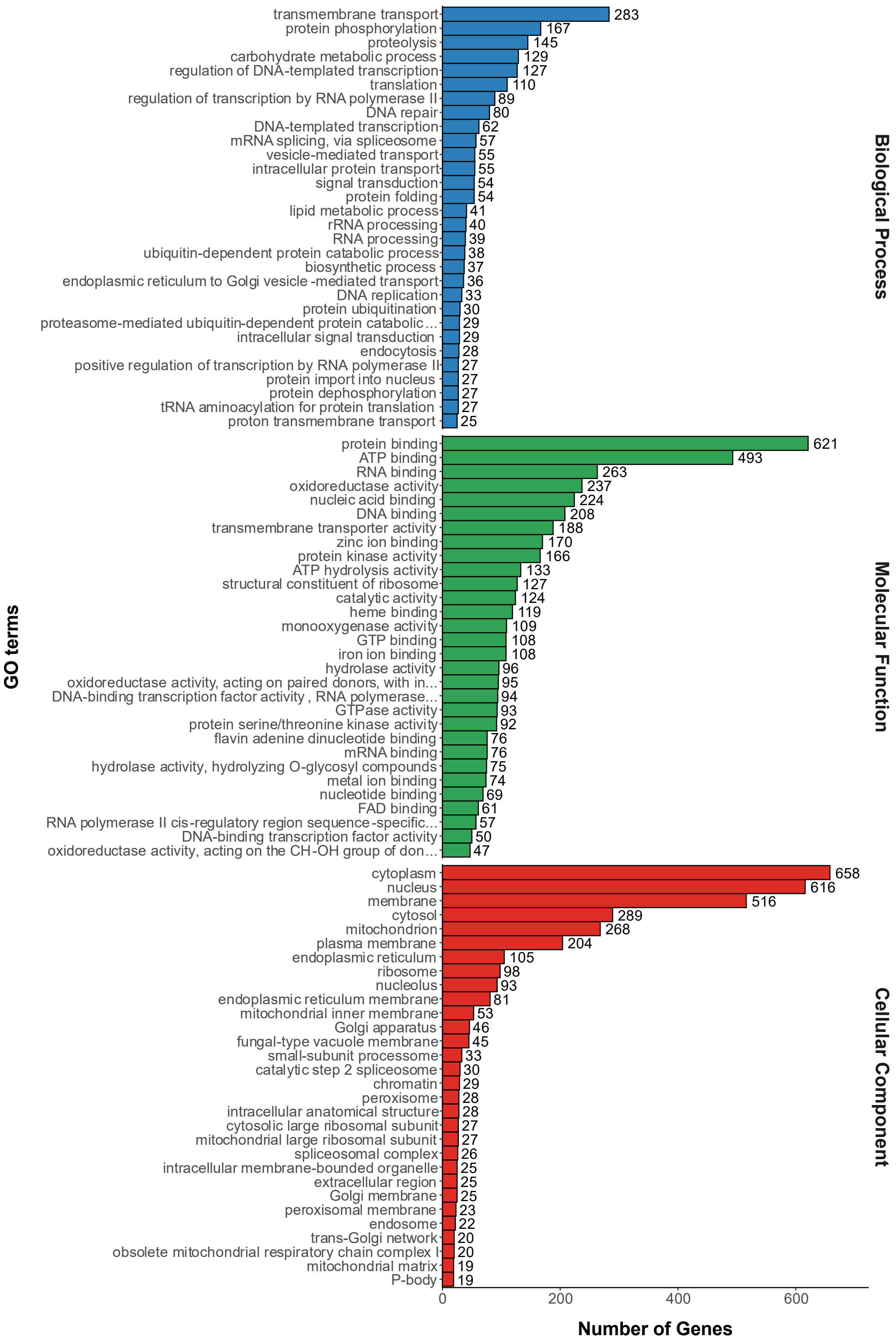

Figure S3. Top 30 gene ontology (GO) terms in each category from the annotated nuclear genome of *Laricifomes officinalis* F01.
